# Pasteurized *Akkermansia muciniphila* protects from fat mass gain but not from bone loss

**DOI:** 10.1152/ajpendo.00425.2019

**Published:** 2020-01-21

**Authors:** Lina Lawenius, Julia M. Scheffler, Karin L. Gustafsson, Petra Henning, Karin H. Nilsson, Hannah Colldén, Ulrika Islander, Hubert Plovier, Patrice D. Cani, Willem M. de Vos, Claes Ohlsson, Klara Sjögren

**Affiliations:** ^1^Centre for Bone and Arthritis Research, Institute of Medicine, Sahlgrenska Academy at University of Gothenburg, Gothenburg, Sweden; ^2^Department of Rheumatology and Inflammation Research, Institute of Medicine, Sahlgrenska Academy at University of Gothenburg, Gothenburg, Sweden; ^3^Université Catholique de Louvain, Louvain Drug Research Institute, WELBIO (Walloon Excellence in Life Sciences and BIOtechnology), Metabolism and Nutrition Research Group, Brussels, Belgium; ^4^Laboratory of Microbiology, Wageningen University, Wageningen, The Netherlands; ^5^Human Microbiome Research Program, Faculty of Medicine, University of Helsinki, Helsinki, Finland

**Keywords:** *Akkermansia*, bone mass, gut microbiota, osteoporosis, probiotic

## Abstract

Probiotic bacteria can protect from ovariectomy (ovx)-induced bone loss in mice. *Akkermansia muciniphila* is considered to have probiotic potential due to its beneficial effect on obesity and insulin resistance. The purpose of the present study was to determine if treatment with pasteurized *Akkermansia muciniphila* (p*Akk)* could prevent ovx-induced bone loss. Mice were treated with vehicle or p*Akk* for 4 wk, starting 3 days before ovx or sham surgery. Treatment with p*Akk* reduced fat mass accumulation confirming earlier findings. However, treatment with p*Akk* decreased trabecular and cortical bone mass in femur and vertebra of gonadal intact mice and did not protect from ovx-induced bone loss. Treatment with p*Akk* increased serum parathyroid hormone (PTH) levels and increased expression of the calcium transporter *Trpv5* in kidney suggesting increased reabsorption of calcium in the kidneys. Serum amyloid A 3 (SAA3) can suppress bone formation and mediate the effects of PTH on bone resorption and bone loss in mice and treatment with p*Akk* increased serum levels of SAA3 and gene expression of *Saa3* in colon. Moreover, regulatory T cells can be protective of bone and p*Akk*-treated mice had decreased number of regulatory T cells in mesenteric lymph nodes and bone marrow. In conclusion, treatment with p*Akk* protected from ovx-induced fat mass gain but not from bone loss and reduced bone mass in gonadal intact mice. Our findings with p*Akk* differ from some probiotics that have been shown to protect bone mass, demonstrating that not all prebiotic and probiotic factors have the same effect on bone.

## INTRODUCTION

Treatment with probiotic bacteria and/or prebiotics can alter the composition of the gut microbiota or its metabolic activity and thereby confer a health benefit to its host (2). Several studies support that different probiotic bacteria can protect from bone loss due to sex steroid deficiency in rodents (1, 6, 20, 27, 31). In a clinical study, daily treatment with a mixture of three *Lactobacillus* strains during 1 yr protected against lumbar spine bone mineral density (BMD) decrease in postmenopausal women (14). Moreover, *Lactobacillus reuteri* was protective of decrease in volumetric BMD in distal tibia in older women (25). Lately, the bacterium *Akkermansia muciniphila* has received attention due to its beneficial effects on metabolic disorders and it is considered to have probiotic potential (11, 33).

In 2012, our group demonstrated that the gut microbiota is a regulator of bone mass in mice (39). We and others have shown that colonized mice have decreased bone mass associated with an altered immune status in bone compared with germ-free mice (20, 26, 28, 39). In contrast, other groups have demonstrated no effect on bone mass or an increased bone mass and growth associated with increased serum levels of IGF-I after colonization of germ-free mice (34, 37). Differences in genetic background and age of the mice, length of colonization, and diet could all be reasons for the conflicting results. In line with this, Yan et al. demonstrated that the short-term effect of the gut microbiota 1 mo after colonization of germ-free mice was a reduction of bone mass and increased bone resorption while long-term colonization increased overall body growth and serum IGF-I, resulting in enhanced longitudinal and radial bone growth (49). In a study by Li et al. (20), sex steroid deficiency increased intestinal permeability in conventionally raised mice but not in germ-free mice. Mice treated with probiotics were protected from increased intestinal permeability and bone loss, suggesting that part of the effect of sex steroid deficiency on bone mass is mediated by increased gut permeability (20). We recently introduced the term “osteomicrobiology” to describe the cross-disciplinary research field where the role of gut microbiota on bone health is studied (29).

A subset of T cells, regulatory T (Treg) cells, can suppress formation and activity of osteoclasts (15, 22, 50). Ovariectomy (ovx) decreases frequency of Treg cells in bone marrow, but this is reversed by probiotic treatment (6, 27). Different strains of the probiotic bacteria *Lactobacillus, Bifidobacterium*, and *Streptococcus* have been shown to differentiate Treg cells both in vitro (17, 30) and in vivo (6, 10, 17, 18, 27). In a recent study, Tyagi et al. showed that treatment of mice with *Lactobacillus rhamnosus* GG increased bone mass and the production of Treg cells in Peyer's patches, spleen, and bone marrow (43). *L. rhamnosus* GG treatment increased serum levels of butyrate that has previously been associated with a protective effect on bone, but interestingly Tyagi et al. showed that the effect of butyrate was dependent on Treg cells (21, 43).

Bone mass is maintained by an adequate calcium supply from diet and uptake from the gut. Improved calcium absorption has been demonstrated in both rats and humans after treatment with prebiotics (46, 47). Low serum concentrations of calcium increase serum parathyroid hormone (PTH), which in turn increases bone resorption and reabsorption of calcium in the kidneys to maintain serum calcium levels.

*A. muciniphila* is a major intestinal species that represents 1–5% of the microbial community in humans (4, 8, 9). Treatment with *A. muciniphila* was previously shown to protect from metabolic disorders, including fat-mass gain, metabolic endotoxemia, adipose tissue inflammation, and insulin resistance when mice were fed a high-fat diet (11). Interestingly, pasteurized *A. muciniphila* (p*Akk*) enhanced protection from metabolic disorders in mice fed high-fat diet (33). Mice treated with p*Akk* also had increased goblet cell number, suggesting increased mucus layer and improved gut barrier function (33). Moreover, p*Akk* was recently evaluated in a clinical trial of individuals with the metabolic syndrome. Treatment with p*Akk* improved insulin sensitivity, reduced insulinemia and plasma total cholesterol, and was demonstrated to be safe and well tolerated (7). The purpose of this study was to determine if treatment with p*Akk* can protect from ovx-induced bone loss in mice by protecting from increased intestinal permeability and thereby reducing inflammation.

## MATERIALS AND METHODS

### 

#### Ovx mouse model and treatment with pasteurized Akkermansia muciniphila.

Twelve-week-old female C57BL/6 mice from Janvier Laboratories (France) were housed in a standard animal facility under controlled temperature (22°C) and photoperiod (12-h light-dark cycle) and had free access to fresh water and pellet diet (Teklad diet 2016, Envigo). The ovx model is included in the FDA guidelines for preclinical and clinical evaluation for agents used for the treatment of postmenopausal osteoporosis (42). Mice were randomized into four groups (*n* = 8–12/group) and treatment with p*Akk* started 3 days before ovx or sham surgery. p*Akk* (2 × 10^8^ colony-forming units/150 µl with 2.5% glycerol in PBS) or vehicle (PBS with 2.5% glycerol) were given by daily oral gavage for 4 wk. p*Akk* was produced as previously described (33). In brief, *A. muciniphila* was pasteurized for 30 min at 70°C, which is a relatively mild pasteurization that limits the denaturation of cellular components (32, 35). A surface protein on *A. muciniphila* was shown in an earlier study to mediate the beneficial effects of both live *A. muciniphila* and p*Akk* (33). The surface protein was stable for temperatures used for the pasteurization. At the end of the study, mice were anesthetized with Ketalar/Dexdomitor vet, bled from the axillary vein, and thereafter killed by cervical dislocation. Tissues for RNA preparation were snap frozen in liquid nitrogen. Bones were excised and fixed in 4% paraformaldehyde. All experimental procedures involving animals were approved by the regional animal ethics committee in Gothenburg, ethics no. 136-2016.

#### Dual-energy X-ray absorptiometry.

BMD of dissected femurs were analyzed using Faxitron UltraFocus dual-energy X-ray absorptiometry (DXA; Faxitron Bioptics, Tuscon, AZ,). Femurs were scanned using X-ray energy of 40 kV and 0.28 mA for 2.53 s with the spatial resolution 24 µm using ×2 geometric magnification. Images were analyzed using the software VISION DXA (Faxitron Bioptics, Tuscon, AZ).

#### High-resolution microcomputed tomography.

High-resolution microcomputed tomography (µCT) analyses were performed using Skyscan 1172 scanner (Bruker MicroCT, Aartselaar, Belgium) as previously described (24). Briefly, femur and vertebra (L5) were imaged with an X-ray tube voltage of 50 kV, a current of 200 µA, and a 0.5-mm aluminum filter. The scanning angular rotation was 180°, and the angular increment was 0.70°. The voxel size was 4.5 µm isotropically. NRecon (version 1.6.9) was used to perform the reconstruction after the scans. In femur, the trabecular bone proximal to the distal growth plate was selected for analyses within a conforming volume of interest (cortical bone excluded) commencing at a distance of 650 µm from the growth plate and extending a further longitudinal distance of 134 µm in the proximal direction. Cortical measurements were performed in the diaphyseal region of the femur starting at a distance of 5.2 mm from the growth plate and extending a further longitudinal distance of 134 μm in the proximal direction. In vertebra, the trabecular bone in the vertebral body caudal of the pedicles was selected for analysis within a conforming volume of interest (cortical bone excluded) commencing at a distance of 5 µm caudal of the lower end of the pedicles and extending a further longitudinal distance of 225 µm in the caudal direction.

#### Gene expression analyses.

RNA from kidney, jejunum, ileum, and colon were isolated using RNeasy Mini QIAcube kit (Qiagen). RNA from the middiaphyseal cortical bone (tibia) was extracted using TRIzol Reagent (Sigma) followed by RNeasy Mini QIAcube kit (Qiagen). Real-time PCR analyses were run using StepOnePlus Real-Time PCR systems (Applied Biosystems). Predesigned probes for *Rankl* (*Tnfsf11*, Mm00441908_m1), *Opg* (*Tnfrsf11b*, Mm00435452_m1), *Trap* (*Acp5*, Mm00475698_m1), *Ctsk* (Mm00484036_m1), *Col1α1* (Mm00801666_g1), *Trpv5* (Mm01166037_m1), *Saa3* (Mm00441203_m1), *Tnfα* (Mm0043258_m1), *IL17A* (Mm00439619_m1), *Ocln* (Mm00500912_m1), *Cldn2* (Mm00516703_s1), *Cldn3* (Mm00515499_s1), and *Cldn15* (Mm00517635_m1) were used from Applied Biosystems. The mRNA abundance of each gene was calculated using the ΔΔCt method, adjusted for expression of 18s ribosomal RNA (4310893E, Applied Biosystems), and presented as percentage of vehicle-treated sham-operated mice.

#### Serum and urine analyses.

Analyses were performed according to manufacturer’s directives for serum and urine calcium using QuantiChrom Calcium Assay Kit (Bioassays systems, Hayward, CA) and urine creatinine using Mouse Creatinine Kit (Crystal Chem, Downers Grove, IL). An EIA kit was used to measure serum 25-hydroxy vitamin D (Immunodiagnostic Systems, Herlev, Denmark) and serum collagen type I COOH-terminal telopeptides (Immunodiagnostics Systems, Herlev, Denmark). ELISA kits were used to measure serum osteocalcin using Mouse Osteocalcin Kit (Immutopics, San Clemente, CA), PTH using Mouse-I-PTH ELISA Kit (Elabscience, Houston, TX), and Mouse SAA-3 ELISA Kit (Millipore, Billerica, MA).

#### Flow cytometry.

Bone marrow cells from one femur and mesenteric lymph nodes cells were isolated and erythrocytes in bone marrow were lysed using 0.83% ammonium chloride. Cells were stained with eBioscience Fixable Viability Dye eFluor 780 according to the manufacturer’s protocol (Invitrogen, ThermoFisher Scientific). Cells were extracellularly stained with anti-CD3-BV510 (Clone 17A2, Nordic BioSite AB, Täby, Sweden), anti-CD4-FITC (Clone RM4-5, Nordic BioSite AB, Täby, Sweden), and anti-CD25-APC (Clone 3C7, BD, Franklin Lakes, NJ). Cells were fixed and permeabilized using the FoxP3 staining buffer kit (Invitrogen, Thermofisher Scientific) and intracellularly stained with anti-Foxp3-PE (Clone FJK-16s, ThermoFisher Scientific) according to manufacturer’s instruction. Treg cells were defined as CD4+CD25+Foxp3+, and results are expressed as frequency of live cells or number of cells. Samples were run on BD FACSVerse (BD, Franklin Lakes, NJ), and data were analyzed using FlowJo software (version 10.4.1).

#### Statistics.

GraphPad Prism (version 7.03) was used for all statistical analyses. Results are presented as means ± SE. Normal distribution was analyzed using the Shapiro-Wilk test. If the sample distribution did not pass the normality test, values were normalized by log transformation before analysis. The overall effect of treatment (Veh/p*Akk*) and surgical procedure (Sham/Ovx) and their interaction were calculated using two-way ANOVA followed by Tukey’s or Dunnett’s post hoc test to correct for multiple comparisons between all groups or all groups versus vehicle-treated sham mice. *P* ≤ 0.05 was considered significant.

## RESULTS

### 

#### Treatment with pasteurized Akkermansia muciniphila reduces fat mass gain.

**Fig. 1.**
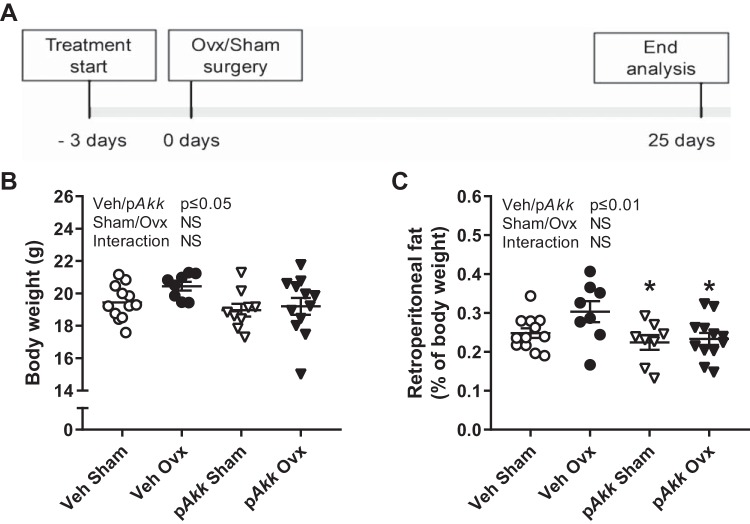
Treatment with pasteurized *Akkermansia muciniphila* reduces fat mass. Outline of study design (*A*). Twelve-week-old female mice were treated with either vehicle (Veh) or pasteurized *Akkermansia muciniphila* (p*Akk*) by daily oral gavage during 28 days, starting 3 days before ovariectomy (Ovx) or sham surgery. At the end of the study, body weight (*B*) and percentage of retroperitoneal fat (*C*) were measured. Values are given as means ± SE (*n* = 8–12). The overall effect of treatment (Veh/p*Akk*) and surgical procedure (Sham/Ovx) and their interaction were calculated using two-way ANOVA followed by Tukey’s post hoc test to correct for multiple comparisons between all groups, *P ≤ 0.05 for p*Akk*-treated sham mice and p*Akk*-treated ovx-mice vs. veh-treated ovx-mice.

Twelve-week-old female mice were treated with either vehicle (veh) or p*Akk* by daily oral gavage during 28 days, starting 3 days before ovx or sham surgery (Fig. 1*A*). Ovx resulted in an expected decrease in uterus weight and increase in thymus weight that was similar in both treatment groups (Table 1). Earlier studies have shown that p*Akk* has beneficial metabolic effects and protects from fat mass accumulation. In the present study p*Akk*-treated mice had decreased body weight and retroperitoneal fat independent of ovx (Fig. 1, *B* and *C*). The percentage of retroperitoneal fat was reduced for both gonadal intact p*Akk*-treated mice (−36%) and p*Akk*-treated ovx-mice (−34%) compared with veh-treated ovx-mice indicating that p*Akk* protects from ovx-induced fat mass accumulation (Fig. 1*C*). Treatment with p*Akk* decreased femur growth indicating a reduced growth of the long bones (Table 2). In contrast, the vertebral height and relative muscle weight were normal in p*Akk*-treated mice (Tables 1 and 3).

**Table 1.** Organ weights

	Surgery		Two-Way ANOVA (*P* Value)		
Treatment	Sham	Ovx	Sham/Ovx	Veh/p*Akk*	Interaction
Uterus, %BW					
Veh	0.332 ± 0.037	0.062 ± 0.004	*P* ≤ 0.01	NS	NS
p*Akk*	0.350 ± 0.070	0.054 ± 0.006			
Thymus, %BW					
Veh	0.219 ± 0.006	0.284 ± 0.009	*P* ≤ 0.01	NS	NS
p*Akk*	0.217 ± 0.016	0.261 ± 0.010			
Kidney, %BW					
Veh	1.022 ± 0.018	1.016 ± 0.019	*P* ≤ 0.05	NS	*P* ≤ 0.05
p*Akk*	1.094 ± 0.034	1.003 ± 0.012			
Liver, %BW					
Veh	4.085 ± 0.092	4.378 ± 0.062	NS	NS	NS
p*Akk*	4.285 ± 0.191	4.365 ± 0.070			
M. quadriceps, %BW					
Veh	0.669 ± 0.031	0.639 ± 0.031	NS	NS	NS
p*Akk*	0.696 ± 0.034	0.631 ± 0.029			

Twelve-week-old female mice were treated with either vehicle (Veh) or pasteurized *Akkermansia muciniphila* (p*Akk*) by daily oral gavage during 28 days, starting 3 days before ovariectomy (Ovx) or sham surgery. At the end of the study tissues were dissected and weighed. Values are given as means ± SE and expressed relative to body weight (BW) (*n* = 8–12). Two-way ANOVA to test the effect of treatment (Veh/p*Akk*) and surgical procedure (Sham/Ovx) and their interaction was used.

**Table 2.** Bone parameters in femur

Treatment	Surgery		Two-Way ANOVA (*P* Value)		
	Sham	Ovx	Sham/Ovx	Veh/p*Akk*	Interaction
Tb N, 1/mm					
Veh	3.26 ± 0.09	3.22 ± 0.15	NS	NS	NS
p*Akk*	3.07 ± 0.18	3.10 ± 0.10			
Tb Sp, mm					
Veh	0.128 ± 0.001	0.126 ± 0.001	NS	NS	NS
p*Akk*	0.128 ± 0.002	128.2 ± 0.001			
Crt Thk, mm					
Veh	0.197 ± 0.002	0.186 ± 0.003	*P* ≤ 0.01	NS	NS
p*Akk*	0.192 ± 0.002	0.184 ± 0.002			
Femur length, mm					
Veh	15.37 ± 0.15	15.61 ± 0.08	NS	*P* ≤ 0.05	NS
p*Akk*	15.19 ± 0.07	15.27 ± 0.13			

Twelve-week-old female mice were treated with either vehicle (Veh) or pasteurized *Akkermansia muciniphila* (p*Akk*) by daily oral gavage during 28 days, starting 3 days before ovariectomy (Ovx) or sham surgery. At the end of the experiment, dissected femurs were analyzed with high-resolution μCT. Trabecular number (Tb N), trabecular separation (Tb Sp), cortical thickness (Crt Thk), and femur length were analyzed. Values are given as means ± SE, (*n* = 8–12). Two-way ANOVA to test the effect of treatment (Veh/p*Akk*) and surgical procedure (Sham/Ovx) and their interaction was used.

**Table 3.** Bone parameters in vertebra

	Surgery		Two-Way ANOVA (*P* Value)		
Treatment	Sham	Ovx	Sham/Ovx	Veh/p*Akk*	Interaction
Tb N, 1/mm					
Veh	5.07 ± 0.11	4.78 ± 0.12	NS	NS	NS
p*Akk*	5.15 ± 0.19	4.95 ± 0.11			
Tb Sp, mm					
Veh	0.150 ± 0.002	0.154 ± 0.003	NS	NS	NS
p*Akk*	0.146 ± 0.004	0.152 ± 0.002			
Crt Thk, mm					
Veh	0.065 ± 0.001	0.056 ± 0.001	*P* ≤ 0.01	NS	NS
p*Akk*	0.060 ± 0.002	0.056 ± 0.001			
Vertebral height, mm					
Veh	3.02 ± 0.02	3.06 ± 0.01	NS	NS	NS
p*Akk*	3.04 ± 0.02	3.06 ± 0.02			

Twelve-week-old female mice were treated with either vehicle (Veh) or pasteurized *Akkermansia muciniphila* (p*Akk*) by daily oral gavage during 28 days, starting 3 days before ovariectomy (Ovx) or sham surgery. At the end of the experiment, dissected vertebras were analyzed with high-resolution microcomputed tomography (µCT). Trabecular number (Tb N), trabecular separation (Tb Sp), and cortical thickness (Crt Thk) were analyzed. Values are given as means ± SE (*n* = 8–12). Two-way ANOVA to test the effect of treatment (Veh/p*Akk*) and surgical procedure (Sham/Ovx) and their interaction was used.

Ovx significantly decreased the relative weights of the kidneys, and this effect was more pronounced in mice treated with p*Akk*, supported by a significant interaction factor (Table 1).

#### Treatment with pasteurized Akkermansia muciniphila reduces bone mass in gonadal intact mice and does not protect against bone loss after ovariectomy.

Several probiotic strains have positive effects on bone, and to determine the possible preventive effect of p*Akk* treatment on ovx-induced bone loss, we measured bone parameters in femur and vertebra by μCT at the end of the study. Dissected femurs were also analyzed by DXA.

**Fig. 2.**
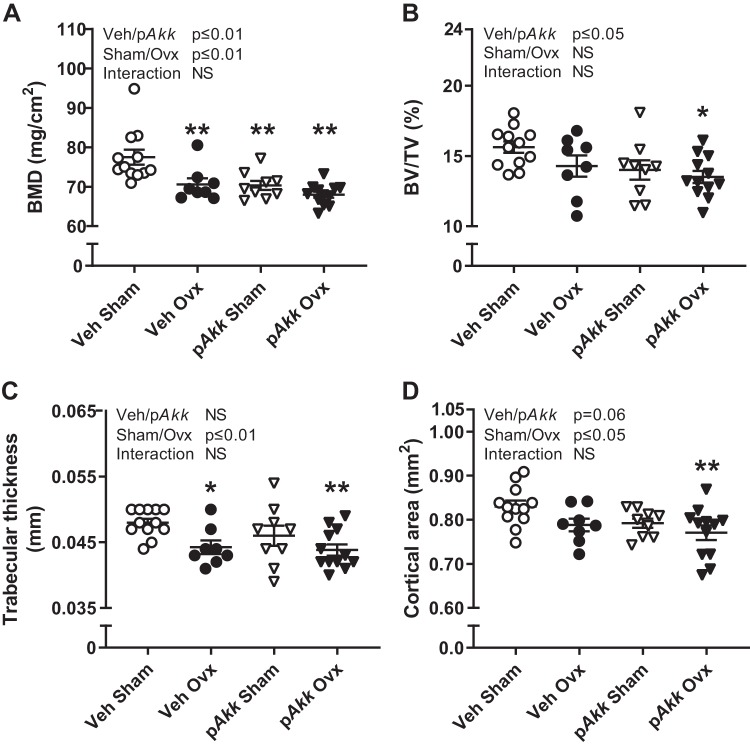
Treatment with pasteurized *Akkermansia muciniphila* reduces bone mass of gonadal intact mice and does not protect against bone loss in femur after ovariectomy (Ovx). Twelve-week-old female mice were treated with either vehicle (Veh) or pasteurized *Akkermansia muciniphila* (p*Akk*) by daily oral gavage during 28 days, starting 3 days before ovx or sham surgery. At the end of the experiment, dissected femurs were analyzed with dual-energy X-ray absorptiometry (DXA) to measure areal bone mineral density (BMD; *A*) and high-resolution microcomputed tomography (μCT) to measure trabecular bone volume fraction (BV/TV; *B*), trabecular thickness (*C*), and cortical area (*D*). Values are given as means ± SE (*n* = 8–12). The overall effect of treatment (Veh/p*Akk*) and surgical procedure (Sham/Ovx) and their interaction were calculated using two-way ANOVA followed by Dunnett’s post hoc test to correct for multiple comparisons between all groups vs. vehicle-treated sham mice, **P ≤ 0.01 and *P ≤ 0.05.

Treatment with p*Akk* did not protect from ovx-induced decrease in areal BMD (Fig. 2*A*), trabecular thickness (Fig. 2*C*), cortical area (Fig. 2*D*), or cortical thickness in femur (Table 2). On the contrary, treatment with p*Akk* decreased areal BMD (Fig. 2*A*) and the trabecular bone volume fraction (BV/TV, Fig. 2*B*). Moreover, there was a tendency (*P* = 0.06) for decreased cortical area when treating mice with p*Akk* (Fig. 2*D*). The gonadal intact mice treated with p*Akk* had significantly decreased areal BMD compared with veh-treated control mice (Fig. 2*A*). The other bone parameters in femur are shown in Table 2.

**Fig. 3.**
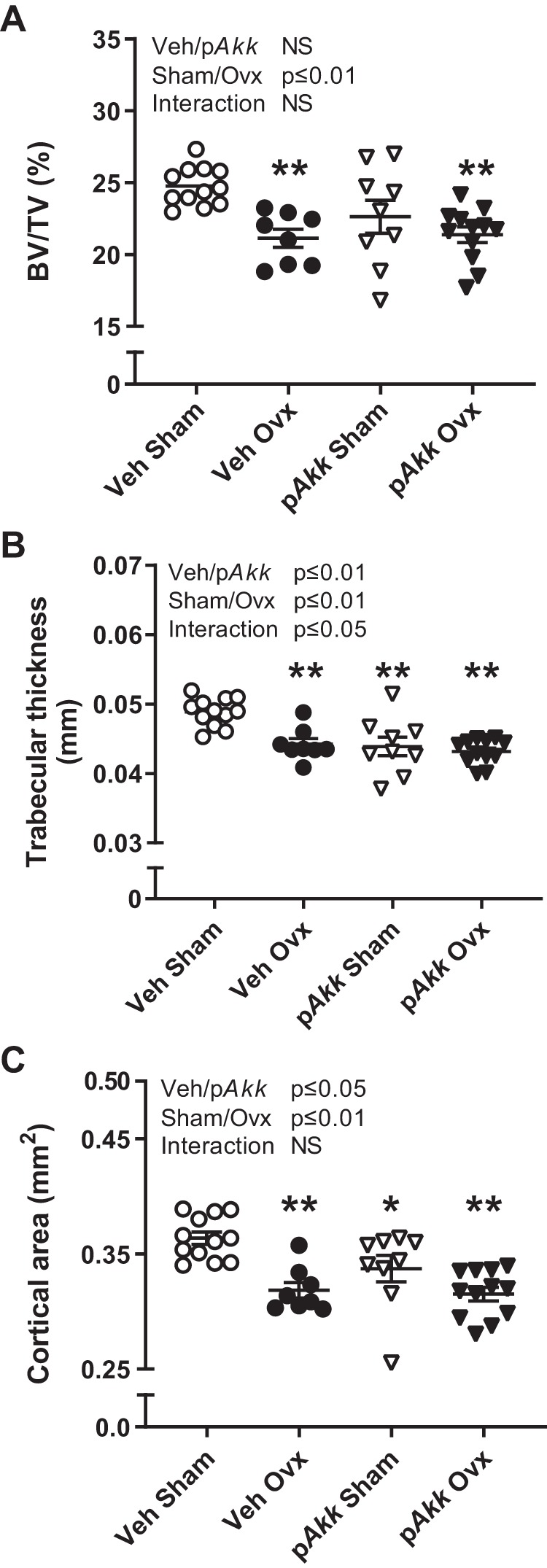
Treatment with pasteurized *Akkermansia muciniphila* reduces bone mass in vertebra of gonadal intact mice and does not protect against bone loss after ovariectomy (Ovx). Twelve-week-old female mice were treated with either vehicle (Veh) or pasteurized *Akkermansia muciniphila* (p*Akk*) by daily oral gavage during 28 days, starting 3 days before ovx or sham surgery. At the end of the experiment, dissected vertebras were analyzed with high-resolution μCT to measure trabecular bone volume fraction (BV/TV; *A*), trabecular thickness (*B*), and cortical (*C*) area. The overall effect of treatment (Veh/p*Akk*) and surgical procedure (Sham/Ovx) and their interaction were calculated using two-way ANOVA followed by Dunnett’s post hoc test to correct for multiple comparisons between all groups vs. vehicle-treated sham mice, **P ≤ 0.01 and *P ≤ 0.05.

In line with the results in femur, treatment with p*Akk* did not protect from ovx-induced bone loss in the vertebra (Fig. 3, *A*–*C*, and Table 3). Furthermore, treatment with p*Akk* resulted in decreased trabecular thickness and cortical area in vertebra of gonadal intact mice compared with veh-treated control mice (Fig. 3, *B* and *C*).

#### Treatment with pasteurized Akkermansia muciniphila increases serum levels of PTH and SAA3 and affects gene expression in cortical bone.

**Fig. 4.**
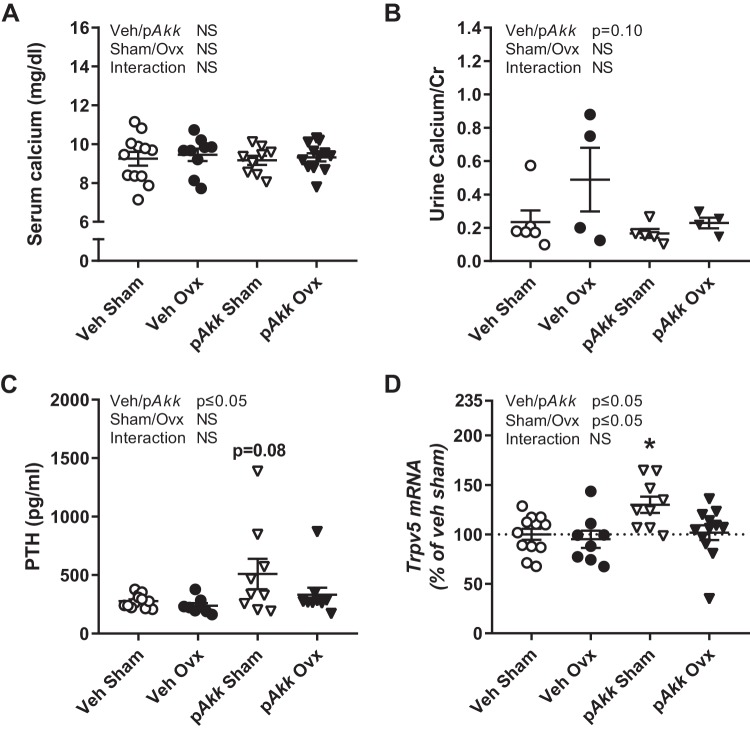
Treatment with pasteurized *Akkermansia muciniphila* increases serum levels of PTH. Twelve-week-old female mice treated with either vehicle (Veh) or pasteurized *Akkermansia muciniphila* (p*Akk*) by daily oral gavage during 28 days, starting 3 days before ovariectomy (Ovx) or sham surgery. Calcium metabolism was analyzed at the end of the experiment by measuring serum levels of calcium (*n* = 8–12; *A*), calcium-creatinine ratio in urine (*n* = 4–6; *B*), serum levels of parathyroid hormone (PTH; *n* = 8–12; *C*), and mRNA expression of transient receptor potential cation subfamily V member 5 (*Trpv5*; *D*) in kidney (*n* = 8–12). Statistical analysis of serum PTH was performed on log-transformed data. The overall effect of treatment (Veh/p*Akk*) and surgical procedure (Sham/Ovx) and their interaction were calculated using two-way ANOVA followed by Dunnett’s post hoc test to correct for multiple comparisons between all groups vs. vehicle-treated sham mice, *P ≤ 0.05.

An adequate calcium supply from the diet and sufficient absorption in the gut is of importance to maintain bone mass. Neither ovx nor p*Akk* treatment affected serum calcium levels (Fig. 4*A*). There was a tendency for decreased calcium/creatinine ratio in urine in mice treated with p*Akk* (*P* = 0.10, Fig. 4*B*). This is in line with the markedly increased PTH levels in p*Akk*-treated mice (Fig. 4*C*). Moreover, p*Akk*-treated gonadal intact mice had a tendency (*P* = 0.08) for increased level of PTH compared with veh-treated gonadal intact mice (Fig. 4*C*). PTH mobilize calcium by increasing bone resorption and reabsorption of calcium in the kidneys. In line with increased serum PTH levels, the mRNA expression of *Trpv5*, a transporter for uptake of calcium in the kidneys, was increased for gonadal intact mice treated with p*Akk* (Fig. 4*D*). The nonactive form of vitamin D, 25 hydroxy vitamin D [25(OH)D_3_], in serum was not affected by p*Akk* treatment but increased with ovx (Table 4). Together these data indicate a decreased calcium absorption from the gut in mice treated with p*Akk*.

**Table 4.** Serum measurements

Treatment	Surgery		Two-Way ANOVA (*P* Value)		
	Sham	Ovx	Sham/Ovx	Veh/p*Akk*	Interaction
25(OH)D_3_, ng/ml					
Veh	41.7 ± 1.2	44.5 ± 1.3	*P* ≤ 0.05	NS	NS
p*Akk*	38.1 ± 2.8	42.9 ± 1.0			
COOH-terminal telopeptides, ng/ml					
Veh	27.2 ± 1.6	43.9 ± 8.0	*P* ≤ 0.05	NS	NS
p*Akk*	31.1 ± 3.2	33.3 ± 2.0			
Osteocalcin, ng/ml					
Veh	96.8 ± 3.8	116.3 ± 8.8	*P* ≤ 0.05	NS	NS
p*Akk*	104.1 ± 8.6	119.4 ± 7.3			

Twelve-week-old female mice were treated with either vehicle (Veh) or pasteurized *Akkermansia muciniphila* (p*Akk*) by daily oral gavage during 28 days, starting 3 days before ovariectomy (Ovx) or sham surgery. At the end of the experiment, serum was analyzed. Values are given as means ± SE (*n* = 8–12). Two-way ANOVA to test the effect of treatment (Veh/p*Akk*) and surgical procedure (Sham/Ovx) and their interaction was used.

**Fig. 5.**
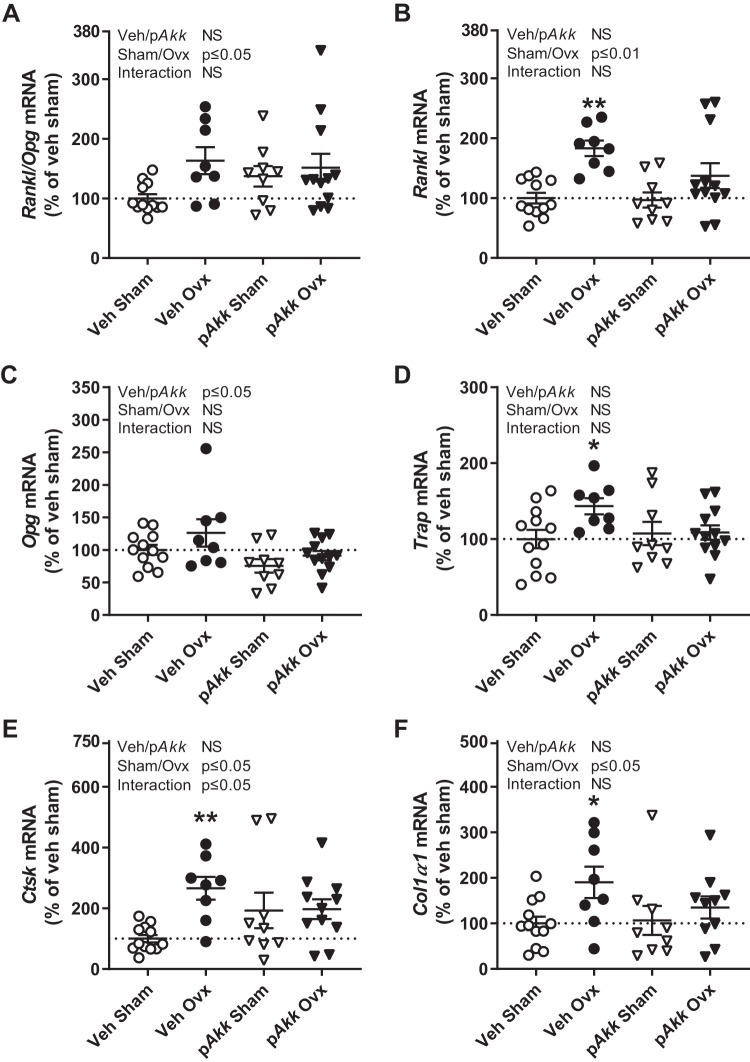
Treatment with pasteurized *Akkermansia muciniphila* reduces *Opg* expression in cortical bone. Twelve-week-old female mice were treated with either vehicle (Veh) or pasteurized *Akkermansia muciniphila* (p*Akk*) by daily oral gavage during 28 days, starting 3 days before ovariectomy (Ovx) or sham surgery. Real-time PCR analysis of gene expression known to affect bone turnover in cortical bone of tibia for ratio of receptor activator of nuclear factor-κB ligand (*Rankl*) and osteoprotegerin (*Opg*; *A*) and individual graphs for *Rankl* (*B*), *Opg* (*C*), tartrate-resistant acid phosphatase (*Trap*; *D*), cathepsin K (*Ctsk*; *E*), and collagen, type I, α1 (*Col1α1*; *F*). Values are given as means ± SE (*n* = 8–12). The overall effect of treatment (Veh/p*Akk*) and surgical procedure (Sham/Ovx) and their interaction were calculated using two-way ANOVA followed by Dunnett’s post hoc test to correct for multiple comparisons between all groups vs. vehicle-treated sham mice, **P ≤ 0.01 and *P ≤ 0.05.

As expected ovx increased bone turnover, reflected both by serum levels of the resorption marker COOH-terminal telopeptides and the formation marker osteocalcin (Table 4). The increased bone turnover was reflected in mRNA expression in cortical bone (Fig. 5, *A*, *B*, *E*, and *F*). The *Rankl*/*Opg* ratio, a major determinant of osteoclastogenesis and bone resorption, was increased by ovx independent of treatment (Fig. 5*A*). Treatment with p*Akk* decreased the expression of *Opg* (Fig. 5*C*).

**Fig. 6.**
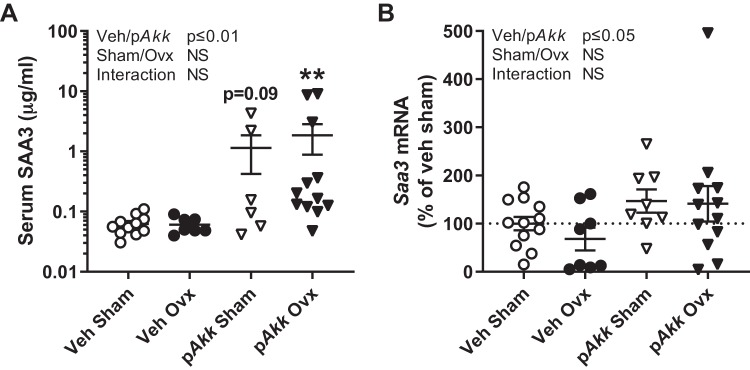
Treatment with pasteurized *Akkermansia muciniphila* increases Serum amyloid A 3 (SAA3) levels in serum and *Saa3* expression in colon. Twelve-week-old female mice were treated with either vehicle (Veh) or pasteurized *Akkermansia muciniphila* (p*Akk*) by daily oral gavage during 28 days, starting 3 days before ovariectomy (Ovx) or sham surgery. SAA3 was analyzed at the end of the experiment by measuring serum levels (*A*) of SAA3 (*n* = 6–12) and mRNA expression (*B*) of *Saa3* in colon (*n* = 8–12). Values are given as means ± SE. Statistical analysis of serum SAA3 was performed on log-transformed data. The overall effect of treatment (Veh/p*Akk*) and surgical procedure (Sham/Ovx) and their interaction were calculated using two-way ANOVA followed by Dunnett’s post hoc test to correct for multiple comparisons between all groups vs. vehicle-treated sham mice, **P ≤ 0.01.

Serum amyloid A (SAA) is a family of apolipoproteins whose levels can rise 1,000-fold in the circulation during inflammation, injury, or infection (44). We originally measured SAA3 as a marker of inflammation to determine the inflammatory effects of ovx and treatment with p*Akk*. Mice treated with p*Akk* had a pronounced increase in serum SAA3 levels and increased *Saa3* expression in colon indicating increased inflammation (Fig. 6, *A* and *B*). However, we observed no differences when treating with p*Akk* in expression of other inflammatory markers (*Tnfα* and *Il17A*) in the intestine (data not shown). Interestingly, SAA3 has been linked with gut microbiota and its production in the colon is associated with the reinforcement of the gut barrier by increasing mucus production signals (41). Accordingly, we found increased *Saa3* expression in colon of mice treated with p*Akk* (Fig. 6*B*). However, SAA3 has also recently been identified as a potential mediator of the effects of PTH to suppress bone formation and increase bone resorption (3). In line with increased serum levels of PTH, we also found that mice treated with p*Akk* had increased serum SAA3 levels (Fig. 6*A*). Moreover, p*Akk*-treated gonadal intact mice had a tendency (*P* = 0.09) for an increased level of SAA3 and p*Akk*-treated ovx-mice had significantly increased levels of SAA3 compared with veh-treated sham mice (Fig. 6*A*).

To evaluate the effect of p*Akk* treatment in the ovx model on gut permeability we analyzed the expression of tight junction proteins (*Ocln*, *Cldn2*, *Cldn3*, and *Cldn15*) in jejunum, ileum, and colon. There were no differences between the groups (data not shown).

#### Treatment with pasteurized Akkermansia muciniphila reduces the frequency of regulatory T cells in the bone marrow.

**Fig. 7.**
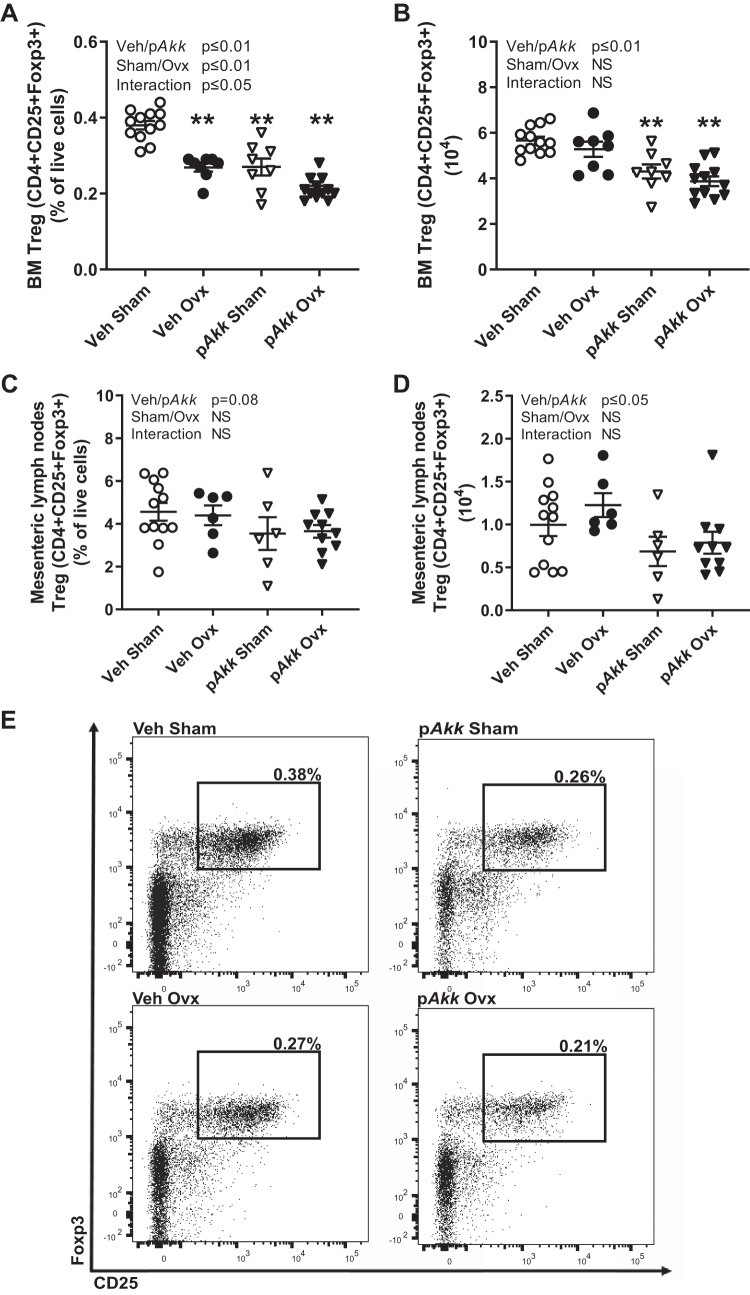
Treatment with pasteurized *Akkermansia muciniphila* decreases the frequency and number of regulatory T cells in bone marrow and number of regulatory T cells in mesenteric lymph nodes. Twelve-week-old female mice treated with either vehicle (Veh) or pasteurized *Akkermansia muciniphila* (p*Akk*) by daily oral gavage during 28 days, starting 3 days before ovariectomy (Ovx) or sham surgery. At the end of the study bone marrow (BM) cells from dissected femur and cells from mesenteric lymph nodes were stained with antibodies recognizing CD4, Foxp3, and CD25. Values represent the frequency of regulatory T cells (Treg, CD4+CD25+Foxp3+) of gated live cells for BM (*A*) and mesenteric lymph nodes (*C*) and absolute number of Treg cells in BM (*B*) and mesenteric lymph nodes (*D*). Representative images of Treg populations in bone marrow from flow cytometry analyses (*E*). Values are given as means ± SE (BM: *n* = 8–12; mesenteric lymph nodes: *n* = 6–12). The overall effect of treatment (Veh/p*Akk*) and surgical procedure (Sham/Ovx) and their interaction were calculated using two-way ANOVA followed by Dunnett’s post hoc test to correct for multiple comparisons between all groups vs. vehicle-treated sham mice, **P ≤ 0.01.

Some probiotic bacteria mediate their anti-inflammatory effects by inducing Treg cells, and the presence of Treg cells can ameliorate bone loss. Earlier studies suggest that Treg cells mediate the beneficial effect of probiotics on bone. Since treatment with p*Akk* caused reduced bone mass in gonadal intact mice and did not protect against bone loss after ovx, we were interested in the effect on Treg cells. Flow cytometry analysis of bone marrow showed that the frequency of Treg (CD4+CD25+Foxp3+) cells in bone marrow was decreased by ovx (Fig. 7, *A* and *E*). Interestingly, treatment with p*Akk* decreased the frequency of Treg cells in the bone marrow also in gonadal intact mice (Fig. 7, *A* and *E*). The decrease was similar in magnitude as the one observed in veh ovx-mice compared with veh sham mice. Since the number of bone marrow cells changes with ovx, we also analyzed the absolute number of Treg cells in bone marrow. Number of Treg cells decreased both in gonadal intact and ovx-mice treated with p*Akk* while there was no effect in veh ovx-mice compared with veh sham mice (Fig. 7*B*). To analyze a lymphoid organ closer to the gut where p*Akk* has its initial effects, we did flow cytometry analysis of cells from mesenteric lymph nodes. There was a tendency (*P* = 0.08) to a decreased frequency and a significantly decreased absolute number of Treg cells in mesenteric lymph nodes for mice treated with p*Akk* (Fig. 7, *C* and *D*).

## DISCUSSION

In the present study, we show that treatment with p*Akk* decreased body weight and retroperitoneal fat mass accumulation. However, treatment with p*Akk* did not protect from ovx-induced bone loss and reduced bone mass in gonadal intact mice. The lower bone mass observed in p*Akk*-treated mice was associated with increased serum levels of PTH and increased expression of the calcium transporter *Trpv5* in kidney and decreased *Opg* expression in cortical bone suggesting increased turnover of calcium. Moreover, the serum level of SAA3 and gene expression of *Saa3* in colon was increased for p*Akk*-treated mice. In addition, the number of Treg cells, which are known to be protective of bone, was decreased in bone marrow and mesenteric lymph nodes after treatment with p*Akk*.

Mice treated with p*Akk* had reduced body weight and retroperitoneal fat mass independent of ovx. This is in line with earlier studies where mice fed a high-fat diet and treated with p*Akk* had reduced fat mass accumulation (33). Mice normally gains body weight after ovx, but this was not observed in the veh-treated ovx-mice compared with veh-treated sham mice. In a study by Li et al., the ovx-mice also lacked body weight gain when treating by biweekly gavage (20). We believe this could be due to stress of the oral gavage.

In contrast to our hypothesis, treatment with p*Akk* did not protect from ovx-induced bone loss but reduced bone mass in gonadal intact mice in both femur and vertebra. The effects of p*Akk* were observed both in trabecular and cortical bone. Moreover, mice treated with p*Akk* had decreased femur length suggesting that treatment with p*Akk* suppressed overall longitudinal bone growth. The femur continues to grow slowly after 3 mo of age until 6 mo of age in mice (12). We have previously shown that treatment with three different probiotic *Lactobacillus plantarum* and *Lactobacillus paracasei* strains protects from ovx-induced bone loss (27). Moreover, the probiotic *Lactobacillus reuteri* protected from ovx-induced bone loss and treatment with the probiotic *L. rhamnosus* GG in sex steroid-deficient mice also protected from bone loss (1, 20). To conclude, different types of bacteria can have completely opposite effects on bone mass. However, it is important to note that in these studies they used live and not pasteurized bacteria.

PTH levels in serum were increased in p*Akk*-treated mice, in line with the observed tendency for decreased calcium/creatinine levels in urine. Furthermore, the mRNA expression of the calcium transporter *Trpv5* in kidney was increased for p*Akk*-treated mice, suggesting increased reabsorption of calcium in the kidneys. The expression of *Trpv5* in the kidneys has previously been shown to be regulated by serum levels of PTH (45). Another mechanism for PTH to mobilize calcium is by promoting bone resorption via increased *Rankl* and decreased *Opg* expression (19, 23). The *Rankl/Opg* ratio is a major determinant of osteoclastogenesis, and the *Rankl/Opg* ratio was increased for ovx-mice, independent of treatment. Interestingly, treatment with p*Akk* decreased *Opg* expression in cortical bone. In contrast, treatment with *L. plantarum* and *L. paracasei* strains was found to increase expression of *Opg* in ovx-mice indicating a completely different mode of action on bone compared with p*Akk* (27). The decrease in *Opg* expression in p*Akk*-treated mice could be due to increased PTH levels and thereby increased resorption. The requirement to mobilize calcium in p*Akk*-treated mice might be a result of decreased calcium uptake in the intestine in response to p*Akk*. *Saa3* expression in colon is associated with increased expression of *Muc2*, the key gene involved in mucus production (41). We found increased *Saa3* expression in colon of mice treated with p*Akk*, suggesting increased mucus production. Earlier studies have demonstrated that live *A. muciniphila* increases the production of mucus and reinforces the gut barrier function (11, 33, 38). Furthermore, the p*Akk*-treated mice had a pronounced increase in serum SAA3 levels, independent of ovx. Recently, it was shown that SAA3 suppresses bone formation and was necessary for the relative increase in bone resorption and bone loss in response to PTH treatment in mice (3). Our results suggest that SAA3 might be the factor mediating the negative impact of p*Akk* on bone. SAA3 is also known to be a marker for inflammation, but we observed no differences when treating with p*Akk* in the expression of other inflammatory markers (*Tnfα* and *IL17A*).

Treatment with probiotic *L. plantarum* and *L. paracasei* strains in ovx-mice has previously shown to protect from decrease in frequency of Treg cells (6, 27). Treg cells suppress osteoclasts and are suggested to be protective of bone (22, 50). In bone marrow, ovx decreased the frequency of Treg cells in line with earlier publications (6, 27). In contrast to treatment with *L. plantarum* and *L. paracasei* strains, p*Akk*-treated mice had decreased frequency of Treg cells. The gonadal intact mice treated with p*Akk* had a similar decrease as the veh-treated ovx-mice. Furthermore, the number of Treg cells was also decreased for p*Akk*-treated mice in bone marrow. The decrease in Treg cells could explain the decrease in bone mass for the mice treated with p*Akk*. The mechanism of how Treg cells are recruited by probiotics is not fully understood, but it has been shown that different probiotic bacteria directly differentiate Treg cells in vitro (17, 30) and induce Tregs in vivo (10, 17, 18, 43). p*Akk* seems to counteract the differentiation of Treg cells. We also analyzed a lymphoid tissue close to the intestine, mesenteric lymph nodes. While ovx showed no effect, a significant decrease in number of Treg cells was observed in p*Akk*-treated mice indicating that p*Akk* also affects Treg-cell differentiation in tissues in close contact with the intestine. Oral administration of *A. muciniphila* has earlier been shown to increase the number of Treg cells (Foxp3+) in adipose tissue of obese mice and in pancreas of diabetic prone mice (13, 38). This may be secondary to the improved glucose tolerance and decreased fat mass accumulation in *A. muciniphila*-treated obese or diabetic prone mice leading to a healthier and less inflamed adipose tissue (48).

In a study by Li et al. (20), germ-free mice were protected from trabecular bone loss induced by sex steroid depletion. Conventionally raised sex steroid-deficient mice lost bone and had increased gut permeability, as well as upregulated osteoclastogenic cytokines in the small intestine and the bone marrow (20). In the same study, ovx-mice treated with the probiotic *L. rhamnosus* GG were protected from increased intestinal permeability and bone loss, which indicates that an improved gut barrier and thereby decreased antigen load passing through the intestinal barrier activating immune cells are mechanisms for the beneficial effect of probiotics on bone. Furthermore, in a study by Schepper et al., treatment with a mucus supplement prevented from increased gut permeability and bone loss caused by antibiotic treatment, indicating a mechanistic link between increased intestinal permeability and bone loss (36). p*Akk*-treated mice fed a high-fat diet were also protected from increased intestinal permeability (33). We therefore hypothesized that treatment of ovx-mice with p*Akk* would decrease gut permeability and protect from bone loss. We found no difference in the expression of tight junction proteins in the gut by ovx or p*Akk* treatment. This is in line with an earlier study that found that the expression of tight junction proteins in the gut following ovx varied in a spatial and temporal manner with no clear effect in either direction (5). A limitation of our study is that we have not measured gut permeability directly. However, opposite to our hypothesis, p*Akk* reduced bone mass in gonadal intact mice and did not prevent from ovx-induced bone loss.

It was previously shown that mice treated with live *A. muciniphila* did not change the composition of the gut microbiota (11). Moreover, neither live *A. muciniphila* nor p*Akk* treatment in humans altered the microbiota composition (7). Furthermore, a systematic review found that six out of seven studies where humans were treated with probiotics had no effect on the microbiota composition (16). Therefore, we did not analyze the composition of the microbiota in this study. Taken together, the effect of either the live bacteria or the pasteurized bacteria is likely due to alterations of metabolic activity of the microbiota and not a change in composition.

To conclude, treatment with p*Akk* reduces the body weight and retroperitoneal fat accumulation in mice. In contrast to our original hypothesis, p*Akk* did not protect from ovx-induced bone loss. p*Akk* reduced bone mass in the gonadal intact mice associated with increased serum levels of PTH and SAA3 and decreased frequency and number of Treg cells in bone marrow, suggesting increased bone resorption. This study does not support a protective role of p*Akk* for bone.

## GRANTS

This study was supported by the Swedish Research Council Grant 2018-02589 and by grants from the Swedish government [under the Avtal om Läkarutbildning och Medicinsk Forskning (Agreement for Medical Education and Research) Grant 238261], Lundberg Foundation Grant 2017-0081, Torsten Söderberg Foundation Grant M65115, and Knut and Alice Wallenberg Foundation Grant KAW 2015.0317. The production of *Akkermansia muciniphila* cells was supported by the unrestricted Spinoza Award of the Netherlands Organization for Scientific Research (to W.M.d.V). P.D.C. is a senior research associate from the FRS-FNRS (Belgium), supported by WELBIO Grant WELBIO-CR-2017C-02) and Funds Baillet Latour (Grant for Medical Research 2015).

## DISCLOSURES

H.P., P.D.C., and W.M.d.V. are inventors of patent applications dealing with the use of *Akkermansia muciniphila* and its components in the context of obesity and related disorders. H.P. is employed by A-Mansia Biotech SA that is commercializing *A. muciniphila*. P.D.C. and W.M.d.V. are co-founders of and have stocks in A-Mansia Biotech SA. C.O. and K.S. are listed as inventors on a patent application regarding the impact of probiotics on bone metabolism and have received research funding for probiotic-related research from Probi AB.

## AUTHOR CONTRIBUTIONS

L.L., J.M.S., H.P., P.D.C., W.M.d.V., C.O., and K.S. conceived and designed research; L.L., J.M.S., K.L.G., P.H., K.H.N., H.C., and K.S. performed experiments; L.L., J.M.S., and K.S. analyzed data; L.L., J.M.S., C.O., and K.S. interpreted results of experiments; L.L. and K.S. prepared figures; L.L., C.O., and K.S. drafted manuscript; L.L., J.M.S., K.L.G., P.H., K.H.N., H.C., U.I., H.P., P.D.C., W.M.d.V., C.O., and K.S. edited and revised manuscript; L.L., J.M.S., K.L.G., P.H., K.H.N., H.C., U.I., H.P., P.D.C., W.M.d.V., C.O., and K.S. approved final version of manuscript.
